# IL-10 control of CD11c^+^ myeloid cells is essential to maintain immune homeostasis in the small and large intestine

**DOI:** 10.18632/oncotarget.8337

**Published:** 2016-03-24

**Authors:** Mathilde J.H. Girard-Madoux, Juliane L. Ober-Blöbaum, Léa M.M. Costes, Junda M. Kel, Dicky J. Lindenbergh, Inge Brouwers-Haspels, Astrid P. Heikema, Janneke N. Samsom, Björn E. Clausen

**Affiliations:** ^1^ Department of Immunology, Erasmus MC, University Medical Center, 3015 GE Rotterdam, Netherlands; ^2^ Institute for Molecular Medicine, University Medical Center of the Johannes Gutenberg-University Mainz, 55131 Mainz, Germany; ^3^ Department of Pediatric Gastroenterology, Erasmus MC, University Medical Center, 3015 GE Rotterdam, Netherlands; ^4^ Department of Medical Microbiology and Infectious Diseases, Erasmus MC, University Medical Center, 3015 GE Rotterdam, Netherlands

**Keywords:** CD11c^+^ myeloid cells, dendritic cells, interleukin 10, small intestine, celiac disease

## Abstract

Although IL-10 promotes a regulatory phenotype of CD11c^+^ dendritic cells and macrophages *in vitro*, the role of IL-10 signaling in CD11c^+^ cells to maintain intestinal tolerance *in vivo* remains elusive. To this aim, we generated mice with a CD11c-specific deletion of the IL-10 receptor alpha (*Cd11c^cre^Il10ra^fl/fl^*). In contrast to the colon, the small intestine of *Cd11c^cre^Il10ra^fl/fl^* mice exhibited spontaneous crypt hyperplasia, increased numbers of intraepithelial lymphocytes and lamina propria T cells, associated with elevated levels of T cell-derived IFNγ and IL-17A. Whereas naive mucosal T-cell priming was not affected and oral tolerance to ovalbumin was intact, augmented T-cell function in the lamina propria was associated with elevated numbers of locally dividing T cells, expression of T-cell attracting chemokines and reduced T-cell apoptosis. Upon stimulation, intestinal IL-10Rα deficient CD11c^+^ cells exhibited increased activation associated with enhanced IL-6 and TNFα production. Following colonization with Helicobacter hepaticus *Cd11c^cre^Il10ra^fl/fl^* mice developed severe large intestinal inflammation characterized by infiltrating T cells and increased levels of *Il17a*, *Ifng*, and *Il12p40*. Altogether these findings demonstrate a critical role of IL-10 signaling in CD11c^+^ cells to control small intestinal immune homeostasis by limiting reactivation of local memory T cells and to protect against *Helicobacter hepaticus*-induced colitis.

## INTRODUCTION

IL-10 is a pleiotropic cytokine with potent anti-inflammatory properties that plays a major role attenuating excessive inflammatory reactions and limiting the magnitude of T-cell responses [1]. IL-10–deficient (*Il10*^−/−^) and IL-10 receptor (IL-10R)–deficient mice develop enhanced T helper (Th) type-1/Th17 responses to intestinal bacterial antigens, which induce severe colitis [2, 3]. When housed under conventional non-SPF conditions *Il10*^−/−^ mice also exhibit small intestinal enteropathy characterized by villus atrophy and crypt hyperplasia, associated with T lymphocyte and IgA^+^ plasma cell infiltration [2]. In an SPF environment *Il10*^−/−^ mice develop minimal intestinal inflammation, while colonization with the intestinal pathogen *Helicobacter hepaticus* elicits severe colitis, demonstrating that specific microbial antigens are pivotal for disease development [4]. Similarly, in humans rare genetic deficiencies in IL-10, IL-10R or its downstream signaling cascade lead to a loss of tolerance to intestinal microbial antigens. Consequently, these patients develop early-onset inflammatory bowel disease (IBD) [5–7]. This prominent role of IL-10 in maintaining intestinal immune homeostasis raises vital questions regarding the precise regulation of IL-10 signaling and its role in different cellular and morphologic compartments within the gastrointestinal tract.

While many cells have the capacity to secrete IL-10, T cell-derived IL-10 is crucial to preserve intestinal homeostasis since T cell-specific *Il10*^−/−^ mice exhibit inflammation in the small intestine (SI) and colon, closely resembling the phenotype of *Il10*^−/−^ mice [8]. At the same time, T cells are thought to be the principal targets of IL-10 regulation [9, 10]. In the colonic lamina propria (LP), IL-10 secretion by CD4^+^Foxp3^+^ regulatory T cells (Treg) is necessary to constrain effector Th1 and Th17 cells [11]. Simultaneously, Foxp3^+^ Treg require IL-10 signals to maintain their regulatory function to prevent colitis [9, 11, 12].

Dendritic cells (DC) govern T-cell function during both primary differentiation in lymphoid organs as well as secondary reactivation in peripheral tissues [13]. *In vitro*, DC cultured in the presence of IL-10 acquire a tolerogenic phenotype and fail to induce primary antigen-specific T-cell responses but rather promote T-cell anergy or the development of Treg [14, 15]. Recently, we have demonstrated that IL-10 also acts on DC *in vivo* and thereby indirectly suppresses effector T-cell responses in the skin [16, 17].

The intestinal LP is densely populated by macrophages and DC both of which contribute to the maintenance of tissue homeostasis and integrity, but appear complementary in function [18–20]. DC have the capacity to migrate to the draining mesenteric lymph nodes (MLN) while macrophages are non-migratory, highly phagocytic and locally maintain Treg [21]. As both DC and macrophages within the LP express CD11c and MHCII their possibly distinct functions are difficult to dissect. On the basis of genetic profiling and cellular precursors CD103^+^CX3CR1^−^ cells within the CD11c^+^MHCII^+^ phagocytes are considered DC [22–25]. These CD103^+^ DC are further divided into CD11b^+^ and CD11b^−^ subsets that have the capacity to migrate to the MLN [25–27]. The exact origin of a third population of CD11c^+^MHCII^+^CD103^−^CD11b^+^ cells in the LP is currently extensively studied. These CD103^−^CD11b^+^ phagocytes express intermediate to high levels of CX3CR1, lie anatomically close to the epithelial barrier, have been detected in the draining lymph and were originally regarded as monocyte-derived DC [22, 26]. However, transcriptional profiling of these CD103^−^CD11b^+^CX3CR1^+^ cells revealed a high similarity to macrophages [22, 28, 29]. Functionally, CD103^−^CD11b^+^CX3CR1^+^ phagocytes appear to exert a dual role by inducing pro-inflammatory Th17 cells and expanding Treg through production of IL-10 [12, 30, 31]. To what extent IL-10 control of CD11c^+^ cells is required to maintain intestinal immune homeostasis is beginning to unfold. Recently, it has been reported that deletion of IL-10Rα expression in CX3CR1^+^ cells renders mice susceptible to spontaneous colitis in a *Helicobacter* positive facility [32]. Moreover, following wild type CD4^+^ T-cell transfer, *Rag2^−/−^* mice lacking both IL-10 and IL-22 signaling develop severe colitis, which cannot be rescued by exogenous IL-10 [33]. Colitis was associated with perturbed Treg cell generation attributed to defective anti-inflammatory macrophage function. In addition, mice with a specific IL-10Rα deletion in macrophages developed no spontaneous colitis in a *Helicobacter* negative facility, but exhibited enhanced susceptibility to transfer colitis and DSS-induced colitis, which was associated with elevated production of TNFα and IL-1β by IL-10Rα-deficient macrophages, leading to enhanced Th17 responses [34, 35]. These data indicate that IL-10 control of phagocytic cells is a key step for the maintenance of intestinal homeostasis. However, it is still unresolved which immune responses, i.e. Th1 and/or Th17, and which mechanisms account for intestinal inflammation in the absence of IL-10 control of myeloid cells and, in particular, whether such regulation is required in both the SI and colon.

In this study, we hypothesized that CD11c^+^ cells constitute critical targets of IL-10 in both the small and large intestine. Using mice with a CD11c-specific deletion of the IL-10Rα (*Cd11c^cre^Il10ra^fl/fl^* mice), we establish that IL-10 control of CD11c^+^ cells is essential to maintain immune homeostasis in the SI by controlling IL-17 and interferon-γ (IFNγ) secreting T cells within the LP. This finding indicates that IL-10 signaling in T cells alone is not sufficient to limit inappropriate T-cell responses in the SI. Upon colonization with *Helicobacter hepaticus Cd11c^cre^Il10ra^fl/fl^* mice develop severe large intestinal disease. Since *Cd11c^cre^Il10ra^fl/fl^* mice exhibit cellular, histological and pathologic features seen in patients with Crohn's and celiac disease, our data strongly suggest harnessing the regulatory function of CD11c^+^ cells to reestablish tolerance in inflammatory intestinal disease.

## RESULTS

### IL-10 signaling in CD11c^+^ cells is required to maintain immune homeostasis in the SI

**Figure 1:**
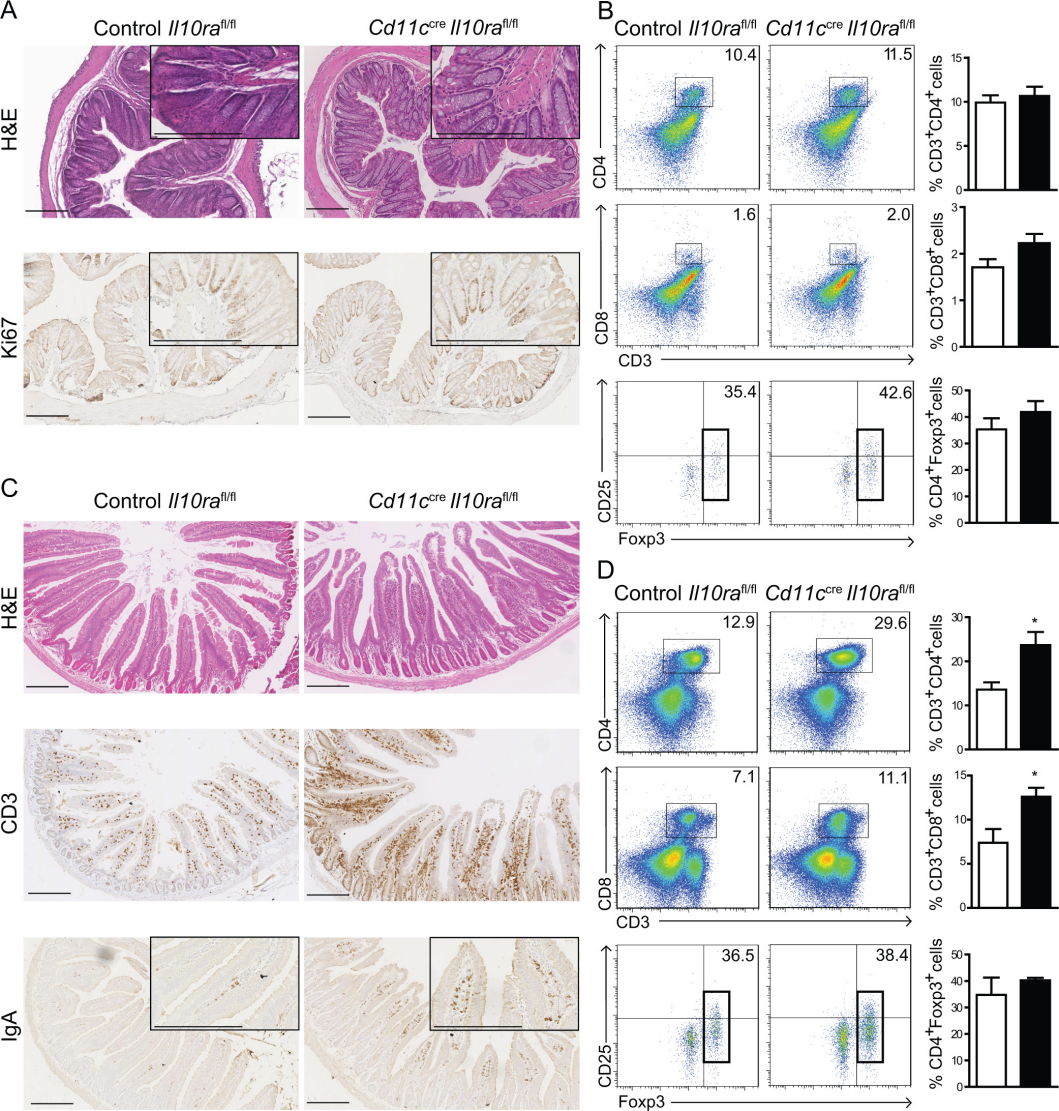
IL-10 control of CD11c^+^ cells maintains small intestinal immune homeostasis (**A**) Paraffin sections of distal colon from 28 week-old control *Il10ra^fl/fl^* and *Cd11c^cre^Il10ra^fl/fl^* littermates were stained with H & E and the proliferation marker Ki67 as indicated. (**B**) Colonic LP cells were isolated and frequencies of CD4^+^ and CD8^+^ T cells as well as CD4^+^Foxp3^+^ Treg were assessed. Gate: CD45^+^ cells. (**C**) Paraffin sections of duodenum from 28 week-old *Cd11c^cre^Il10ra^fl/fl^* and control Il10ra*^fl/fl^* littermates were stained with H & E and anti-CD3 and anti-IgA antibodies as indicated. (**D**) Frequencies of LP T cell subsets from the SI were measured. Gate: CD45^+^B220^−^. (A, C) One representative animal out of 12 is depicted. Bar represents 200 μm. (B, D) *n* = 4 mice per group, 1 out of 3 experiments shown. Data are represented as mean ± SEM. □ Control *Il10ra^fl/fl^, ■ Cd11c^cre^Il10ra^fl/fl^* mice.

When housed in individually ventilated cages (IVC) under SPF conditions *Cd11c^cre^Il10ra^fl/fl^* animals exhibited swollen MLN, but neither developed a prolapse nor any signs of colonic inflammation. In 28 week-old mice, the general morphology of the colon was comparable to Cre-negative *Il10ra^fl/fl^* control mice without any noticeable increase in cellularity or evidence of significant lymphocyte infiltration (Figures 1A and S1A). Cell proliferation in the colonic LP and crypts was not altered as determined with the mitosis marker Ki67 (Figure 1A). The frequencies of CD4^+^ and CD8^+^ T cells, CD4^+^Foxp3^+^ Treg as well as B cells were similar to control *Il10ra^fl/fl^* mice (Figures 1B and S1B).

In contrast, the morphology of the SI in 28 week-old *Cd11c^cre^Il10ra^fl/fl^* mice was severely disturbed. The villi appeared enlarged and LP cellularity was increased containing more CD3^+^ T cells than controls (Figure 1C). While both CD4^+^ and CD8^+^ cells were more frequent, CD4^+^Foxp3^+^ Treg were present at the same frequency as in *Il10ra^fl/fl^* controls (Figure 1D). Similar to *Il10*^−/−^ mice [2], IgA^+^ plasmablasts were more abundant in the LP of *Cd11c^cre^Il10ra^fl/fl^* animals (Figure 1C). These morphological and cellular alterations were much less pronounced in younger mice (Figure S2). Taken together, these data demonstrate that under SPF conditions, IL-10 signaling in CD11c^+^ cells is crucial to maintain immune homeostasis in the SI, but appears less critical in the colon.

### Enhanced crypt activity and numbers of inducible iELs in the SI of *Cd11c^cre^Il10ra^fl/fl^* mice

**Figure 2:**
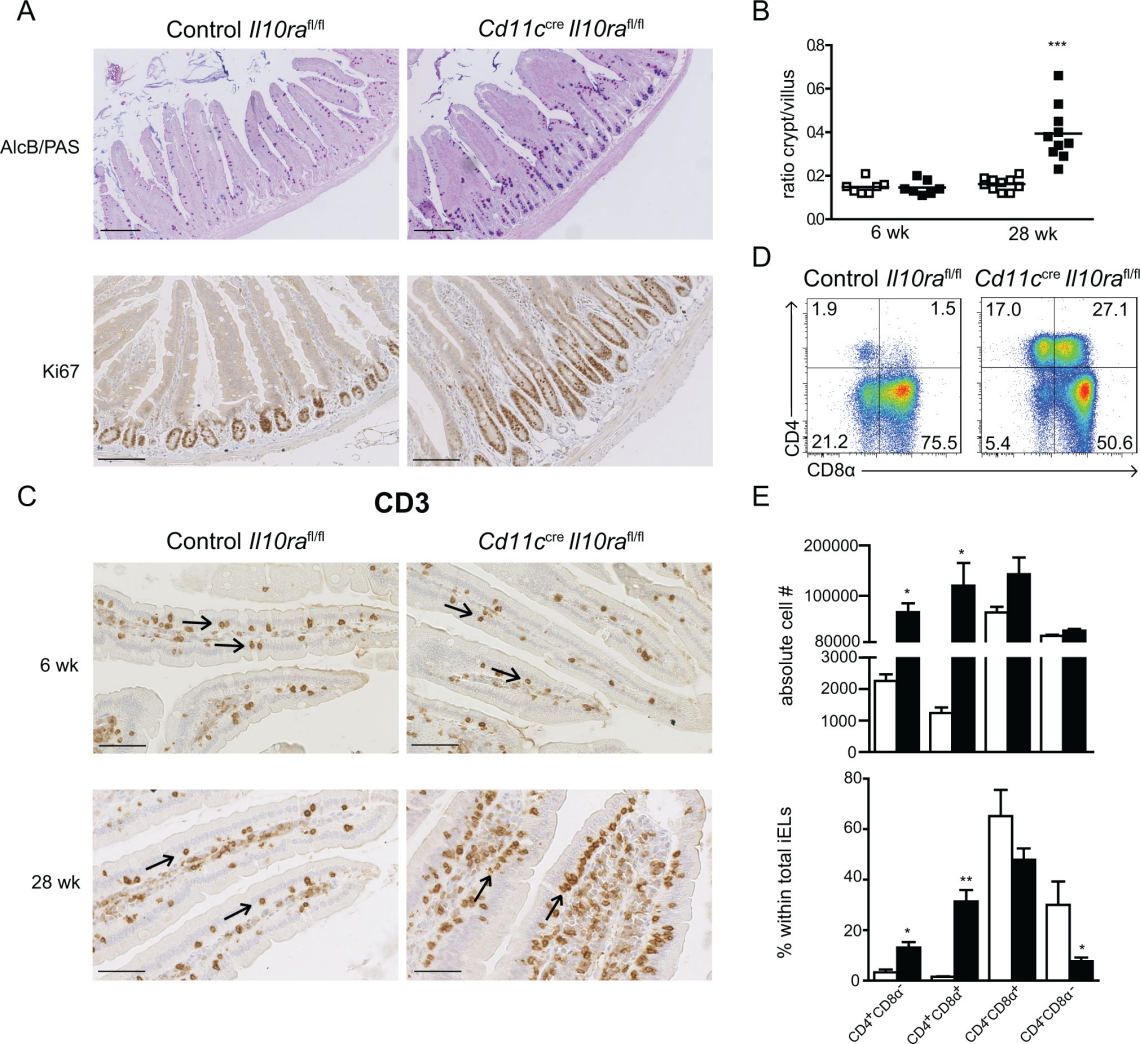
*Cd11c^cre^Il10ra^fl/fl^* mice develop age-related crypt hyperplasia and increased numbers of iELs in the SI (**A**) Duodenum sections of 28 week-old control *Il10ra^fl/fl^* and *Cd11c^cre^Il10ra^fl/fl^* littermates were stained with Alcian blue and PAS to reveal mucus-producing cells and with anti-Ki67 antibody to identify proliferating cells as indicated. Bar represents 200 μm. (**B**) The ratio between crypt and villus length was determined in 6 and 28 week-old mice. Two to 4 crypt/villus ratios of 5 representative mice of each group were measured. Each square represents one crypt/villus ratio. (**C**) Sections of duodenum of 6 and 28 week-old control *Il10ra^fl/fl^* and *Cd11c^cre^Il10ra^fl/fl^* mice were stained with anti-CD3 antibody. iELs are indicated by arrows. Bar represents 50 μm. (**D**–**E**) 28 week-old mice were gavaged twice with 50% EtOH to transiently disrupt the epithelial barrier. (D) One day later mice were sacrificed and iELs were isolated and stained for CD4 and CD8α surface markers. Gate: CD45^+^ cells. (E) Absolute cell number and frequency of iEL populations. *n* = 3 mice per group, 1 out of 4 experiments is depicted. Data are represented as mean ± SEM. □ Control *Il10ra^fl/fl^*, ■ *Cd11c^cre^Il10ra^fl/fl^* mice. (A, C, D) One representative animal out of 12 is depicted.

To establish a possible role of IL-10–mediated regulation of CD11c^+^ cells in the maintenance of epithelial homeostasis, intestinal tissue was analyzed by histology. In the SI crypt hyperplasia is a general sign of inflammation, which is not only observed in *Il10*^−/−^ mice [2], but also in patients with celiac disease or IBD [36, 37]. In 28 week-old *Cd11c^cre^Il10ra^fl/fl^* animals, numerous Ki67^+^ cells were found in elongated crypts (Figure 2A) and consequently, the crypt/villus ratio was significantly higher as compared to controls (Figure 2B). In contrast, at 6 weeks the crypt/villus ratio was similar in control *Il10ra^fl/fl^* and *Cd11c^cre^Il10ra^fl/fl^* mice (Figure 2B). In addition, mucus-producing cells were more frequent in the crypts of 28 week-old *Cd11c^cre^Il10ra^fl/fl^* animals, whereas fewer differentiated cells could be observed along the villi as visualized by Alcian Blue (AlcB)/PAS staining (Figure 2A). These findings indicate that in the absence of IL-10 signaling in CD11c^+^ cells the mice gradually develop small intestinal inflammation associated with disrupted crypt-villus architecture and disturbed epithelial differentiation.

Intraepithelial lymphocytes (iELs) are lining the epithelium and serve as sentinels to maintain epithelium integrity and prevent the entry and spreading of pathogens. They exert both a protective regulatory role through IL-10 secretion and a cytotoxic function by eliminating damaged or infected cells. Increased numbers of iELs are a hallmark of celiac disease [38] and are also observed in some individuals with Crohn's disease [39]. Thus, we sought to determine whether the lack of IL-10 control of CD11c^+^ cells triggered the accumulation of iELs in the SI. Visualization of CD3^+^ cells by immuno-histochemistry demonstrated that in 6 week-old mice, the number of iELs was comparable to *Il10ra^fl/fl^* littermates, whereas at 28 weeks of age *Cd11c^cre^Il10ra^fl/fl^* mice harbored increased numbers of iELs (Figure 2C).

We hypothesized that compromising the epithelial barrier integrity may further enhance the number of CD4^+^ iELs and LP T cells. In wild type mice, ethanol (EtOH) gavage disrupts the epithelial barrier and thereby augments antigen exposure without inducing mucosal inflammation in the SI [40, 41]. Therefore, *Cd11c^cre^Il10ra^fl/fl^* and control *Il10ra^fl/fl^* mice were gavaged with 50% EtOH on two consecutive days and sacrificed the next day. Ethanol gavage increased the percentage of LP CD4^+^ T cells in *Cd11c^cre^Il10ra^fl/fl^* mice (Figure S3A). In addition, the absolute number and frequency of the two populations of CD4^+^ iELs (CD4^+^CD8α^−^ and CD4^+^CD8α^+^) were significantly elevated (Figure 2D and 2E). The higher numbers of both LP T cells and iELs in *Cd11c^cre^Il10ra^fl/fl^* mice after EtOH gavage demonstrate that IL-10 control of CD11c^+^ cells in the SI is required to regulate CD4 T cells upon loss of barrier integrity.

### Elevated levels of T cell-derived pro-inflammatory cytokines in *Cd11c^cre^Il10ra^fl/fl^* mice

**Figure 3:**
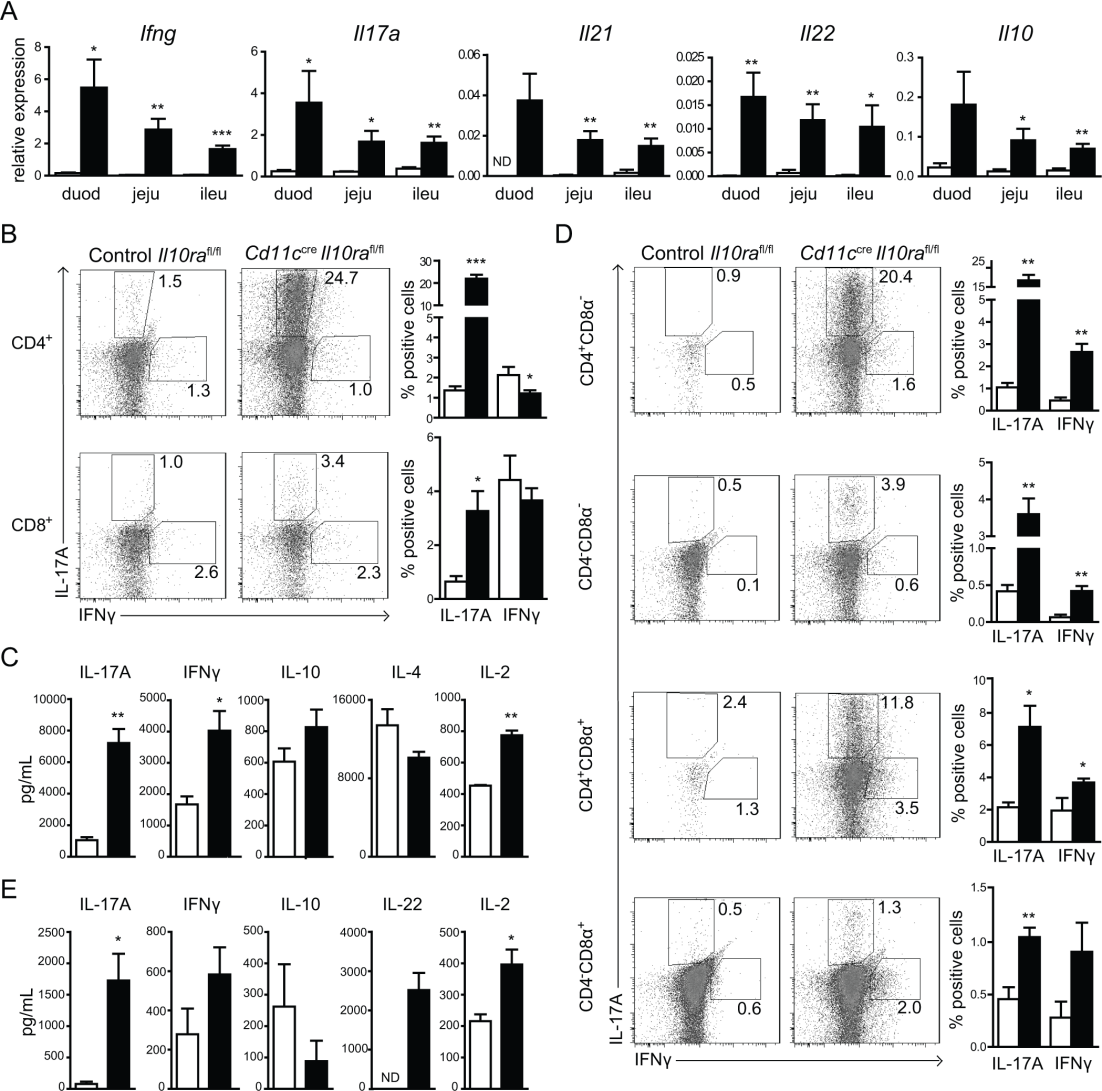
Lack of IL-10 signaling in CD11c^+^ cells leads to enhanced T cell activation in the SI (**A**) mRNA expression levels of several T cell-derived cytokines and anti-inflammatory IL-10 were measured in sections of duodenum (duod), jejunum (jeju) and ileum (ileu) from *Cd11c^cre^Il10ra^fl/fl^* and control *Il10ra^fl/fl^* littermates as indicated. mRNA expression is relative to GAPDH. One out of 2 experiments is shown, *n* = 6 mice per group. (**B**–**E**) Mice were gavaged on day 0 and 1 with 50% EtOH to increase epithelial permeability and sacrificed on day 2. (B) LP cells were isolated and stimulated with PMA and ionomycin for 6 h and subsequently stained for intracellular IL-17A and IFNγ. CD4^+^ and CD8^+^ T cells were analyzed as depicted. (C) Amounts of secreted cytokines were measured in supernatants of *in vitro* stimulated LP cells. (D) iELs were stimulated with PMA and ionomycin for 6 h and stained for surface CD4 and CD8α and intracellular cytokines. Each of the four iEL populations is depicted. (E) Cytokine production by *in vitro* stimulated iELs was measured in the supernatants. One out of 4 experiments is presented, *n* = 3–5 mice per group. Data are represented as mean ± SEM. □ Control *Il10ra^fl/fl^*, ■ *Cd11c^cre^Il10ra^fl/fl^* mice.

The increased T-cell activation induced by IL-10Rα-deficient CD11c^+^ cells could be restricted to a particular subset due to preferential polarization of CD4^+^ Th1, Th17 and/or Th2 cells. Therefore, we next assessed the expression levels of T cell-specific cytokines in the duodenum, jejunum and ileum using quantitative RT-PCR. In contrast to control *Il10ra^fl/fl^* mice, the Th1 and Th17 signature cytokines *Ifng*, *Il17a*, *Il21* and *Il22* were highly expressed in all parts of the SI of *Cd11c^cre^Il10ra^fl/fl^* mice, with a maximum response in the duodenum and gradually decreasing towards the most distal part of the SI (Figure 3A). Likewise, mRNA levels of *Il10* were elevated as compared to controls, while Th2-derived *Il4* was not detectable by RT-PCR. These results indicate that IL10Rα-deficient CD11c^+^ cells selectively induce an inappropriate Th1/Th17 response in the SI despite the augmented presence of IL-10 *in situ*.

To determine whether the increased cytokine expression was associated with higher numbers of Th1/Th17 cells LP cells were isolated from mice two days after EtOH gavage and restimulated with PMA/ionomycin for 6 h *in vitro*. Only 1.4±0.2% (mean±SEM) of control *Il10ra^fl/fl^* CD4^+^ T cells expressed IL-17A, whereas 22.0 ± 1.7% of the cells isolated from *Cd11c^cre^Il10ra^fl/fl^* mice were IL-17A^+^ (Figure 3B). Similarly, three times more LP CD8^+^ T cells expressed IL-17A in the cultures of *Cd11c^cre^Il10ra^fl/f^* animals. In contrast, the frequency of IFNγ-producing CD4^+^ and CD8^+^ T cells was comparable between *Cd11c^cre^Il10ra^fl/f^* mice and *Il10ra^fl/fl^* controls (Figure 3B). In accordance, we detected elevated LP T cell-derived pro-inflammatory cytokines in the supernatants of *Cd11c^cre^Il10ra^fl/fl^* as compared to control cells. The level of IL-17A release was 7-fold increased, and the cells produced twice as much IFNγ and IL-2 (Figure 3C). In contrast, secretion of Th2-derived IL-4 was similar to controls. Taken together these data demonstrate a significant expansion and hyper-activation of Th1 and Th17 cells in the LP of the SI of *Cd11c^cre^Il10ra^fl/fl^* mice. Intriguingly, secretion of anti-inflammatory IL-10 was not altered upon restimulation of *Cd11c^cre^Il10ra^fl/fl^* as compared to control *Il10ra^fl/fl^* LP cells, indicating that IL-10 signaling in T cells alone was not sufficient to prevent excessive Th1/Th17 activation (Figure 3C).

We next investigated the phenotype of iELs after *in vitro* stimulation with PMA/ionomycin. Within all four populations of iELs purified from *Cd11c^cre^Il10ra^fl/fl^* animals the frequency of both IL-17A– and IFNγ-producing cells was significantly increased in comparison to control *Il10ra^fl/fl^* mice (Figure 3D). Similarly to cultures of LP T cells, 15 times more IL-17A and two times more IFNγ and IL-2 were measured in the supernatants of iEL cultures from *Cd11c^cre^Il10ra^fl/fl^* animals (Figure 3E). The Th17 cytokine IL-22 was only produced by iELs isolated from *Cd11c^cre^Il10ra^fl/fl^* mice and there was a trend towards elevated IFNγ and reduced IL-10 secretion. In contrast to LP T cells, no IL-4 could be detected in the iEL culture supernatants. In summary, these data strongly suggest that CD11c^+^ cells need to be controlled by IL-10 in order to prevent excessive activation of LP T cells and iELs. Moreover, these findings indicate a selective enhancement of both Th1 and Th17 responses by IL-10Rα-deficient CD11c^+^ cells.

### IL-10 signaling in CD11c^+^ cells is dispensable during a primary T cell response

**Figure 4:**
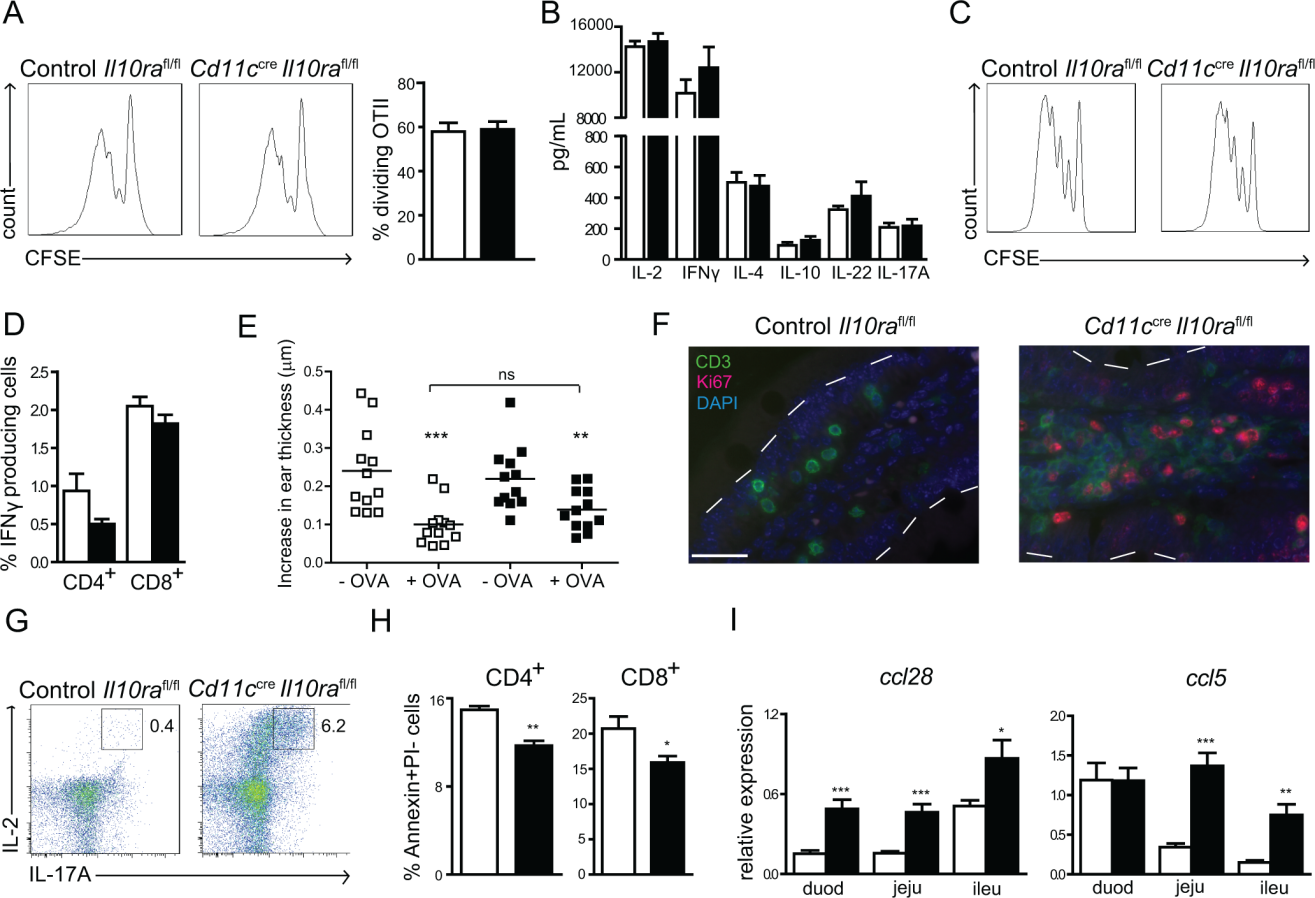
IL-10 control of CD11c^+^ cells is crucial to restrain effector/memory T cell responses in the LP of the SI (**A**) OVA-pulsed MLN CD11c^+^ cells were co-cultured with CFSE-labeled OTII cells for 3 days. T cell-proliferation was assessed by CFSE dilution and (**B**) cytokine secretion was measured in the culture supernatants. One out of 3 experiments with *n* = 6 mice per group is shown. (**C**) On day 0, 10^7^ CFSE-labeled OTII cells were injected i.v. into recipient mice, which were gavaged on day 1 with 70 mg OVA and sacrificed on day 4. Proliferation of OTII T cells in the MLN was measured by CFSE dilution. The experiment was performed twice with *n* = 5 mice per group. (**D**) Mice were gavaged on day 0 and 1 with 50% EtOH and sacrificed on day 2. MLN cells were re-stimulated *in vitro* with PMA/ionomycin and intracellular IFNγ production was determined by flow cytometry. One out of 3 experiments with *n* = 4 mice is shown. (**E**) Oral tolerance to OVA was induced by gavage of 25 mg OVA per mouse. Tolerance was assessed by monitoring a DTH response in the ear as described in the Supplementary Methods section. One out of 2 experiments is depicted. (**F**) Mice were gavaged on day 0 and 1 with 50% EtOH to increase epithelial permeability and sacrificed on day 2. Staining with fluorescently labeled anti-CD3 (green) and anti-Ki67 (red) was performed on duodenum sections from 28 week-old *Cd11c^cre^Il10ra^fl/fl^* and control *Il10ra^fl/fl^* mice. Nuclei were counterstained with DAPI. One representative out of 6 mice is depicted. Bar represents 25 μm. (**G**) LP cells were isolated and re-stimulated *in vitro* with PMA/ionomycin for 6 h. Intracellular FACS staining was performed for IL-2 and IL-17A. One representative out of 6 mice is shown. (**H**) LP cells were isolated and AnnexinV/PI staining was used to assess T cell apoptosis. Gate: CD45^+^ cells. One out of 2 experiments is shown with *n* = 4 mice per group. (**I**) Expression of *Ccl28* and *Ccl5* mRNA was determined by quantitative RT-PCR in duodenum (duod), jejunum (jeju) and ileum (ileu) of *Cd11c^cre^Il10ra^fl/fl^* and control *Il10ra^fl/fl^* littermates. mRNA expression is relative to GAPDH. *n* = 6 mice, one out of 2 experiments is depicted. Data are represented as mean ± SEM. □ Control *Il10ra^fl/fl^*, ■ *Cd11c^cre^Il10ra^fl/fl^* littermate mice.

The excessive Th1/Th17 activation in the SI of *Cd11c^cre^Il10ra^fl/fl^* mice could be the result of either enhanced priming of antigen-specific naive T cells in the MLN or increased reactivation of memory T cells in the LP. To determine whether IL-10 signaling in CD11c^+^ cells is crucial during initiation of a primary response, CD11c^+^ cells were isolated from MLN, pulsed with ovalbumin (OVA) peptide and incubated with OVA-specific CFSE-labeled OTII T cells. After 3 days, OTII proliferation was similar irrespective of whether the T cells were instructed by *Cd11c^cre^Il10ra^fl/fl^* or control *Il10ra^fl/fl^* cells (Figure 4A). In accordance, levels of pro- and anti-inflammatory cytokines in the supernatants of these cultures were comparable (Figure 4B). To confirm that naive T-cell priming was also not affected *in vivo*, we injected CFSE-labeled OTII cells from Ly5.1 donors into *Cd11c^cre^Il10ra^fl/fl^* and control *Il10ra^fl/fl^* Ly5.2 recipient mice. Animals were fed with OVA protein the next day and sacrificed 72 h later. Cells were isolated from MLN and OTII cells were analyzed by flow cytometry for CFSE dilution. OTII cells proliferated similarly in *Cd11c^cre^Il10ra^fl/fl^* mice and controls (Figure 4C). To dissect the cytokines expressed by primed T cells in the MLN, mice were gavaged on day 0 and 1 with 50% EtOH and sacrificed on day 2. After *in vitro* stimulation of MLN cells with PMA/ionomycin for 4–6 h, we performed intracellular cytokine staining for IFNγ, IL-17A and IL-4 to determine naïve T-cell skewing by flow cytometry. While similar percentages of both CD4^+^ and CD8^+^ IFNγ-producing T cells were induced in *Cd11c^cre^Il10ra^fl/fl^* and control mice (Figure 4D), T cells secreting either IL-17A or IL-4 were not detectable. These data indicate that IL-10 signaling in CD11c^+^ cells does not influence naïve T-cell priming. Hence, we hypothesized that oral tolerance to harmless antigens, which is dependent on antigen presentation by DC and subsequent differentiation of Treg in the MLN [42, 43], would still be intact in the presence of IL-10 signaling deficient CD11c^+^ cells. Therefore, *Cd11c^cre^Il10ra^fl/fl^* mice were subjected to a classical oral tolerance induction protocol and delayed type hypersensitivity response. As depicted in Figure 4E, *Cd11c^cre^Il10ra^fl/fl^* mice develop oral tolerance to the same extent as control mice. These results demonstrate that T-cell differentiation in the MLN is not affected by lack of IL-10R signaling in CD11c^+^ cells and strongly suggest that defective IL-10 signaling in CD11c^+^ cells results in enhanced effector/memory T cell responses via local deregulation in the LP.

### Enhanced proliferation, survival and recruitment of LP cells in the SI of *Cd11c^cre^Il10ra^fl/fl^* mice

Enhanced reactivation of effector/memory cells could be the result of increased proliferation, diminished apoptosis and/or enhanced recruitment into the LP (T cells and IgA^+^ plasmablasts) and epithelium (iELs). First, double staining with anti-CD3 and anti-Ki67 revealed very few LP CD3^+^ cells co-expressing nuclear Ki67 in control *Il10ra^fl/fl^* mice. In contrast, a large number of CD3^+^Ki67^+^ actively mitotic cells was present in *Cd11c^cre^Il10ra^fl/fl^* animals (Figure 4F), which was already detectable without disruption of the epithelium by EtOH (Figure S3B) and could be confirmed using bromodeoxyuridine (BrdU) incorporation (Figure S3C). Moreover, after PMA/ionomycin restimulation of LP T cells isolated from *Cd11c^cre^Il10ra^fl/fl^* mice, approximately 20% of IL-17A^+^ cells co-expressed IL-2 (6% of total CD4^+^ cells) (Figure 4G), a cytokine critical for Th17 cell expansion [44]. This demonstrates that IL-17A producing T cells in *Cd11c^cre^Il10ra^fl/fl^* animals are more numerous and highly proliferative. Second, we assessed expression of the apoptosis marker Annexin V on LP T cells. Significantly fewer LP CD4^+^ and CD8^+^ cells isolated from *Cd11c^cre^Il10ra^fl/fl^* mice expressed Annexin V on their surface as compared to control *Il10ra^fl/fl^* mice (Figure 4H). Third, to reveal whether recruitment of T cells and IgA^+^ plasmablasts into the LP was enhanced in *Cd11c^cre^Il10ra^fl/fl^* mice, we measured relevant cell-specific chemokines by quantitative RT-PCR. The chemokine CCL28 (MEC) preferentially attracts IgA^+^ cells that are numerous in the LP of *Cd11c^cre^Il10ra^fl/fl^* mice (Figure 1C) as well as celiac disease patients [36]. CCL5 (Rantes) is a T cell-specific chemo-attractant that is elevated in patients with IBD [45]. Expression of both chemokines was significantly increased across all parts of the SI of *Cd11c^cre^Il10ra^fl/fl^* animals, except for the levels of *Ccl5* in the duodenum (Figure 4I). In conclusion, these data demonstrate that IL-10 signaling in CD11c^+^ cells is essential to prevent uncontrolled reactivation of small intestinal LP memory T cells *in situ* by multiple mechanisms, involving T cell proliferation, apoptosis and chemo-attraction.

### IL10Rα-deficient CD11c^+^ cells produce elevated levels of pro-inflammatory cytokines

**Figure 5:**
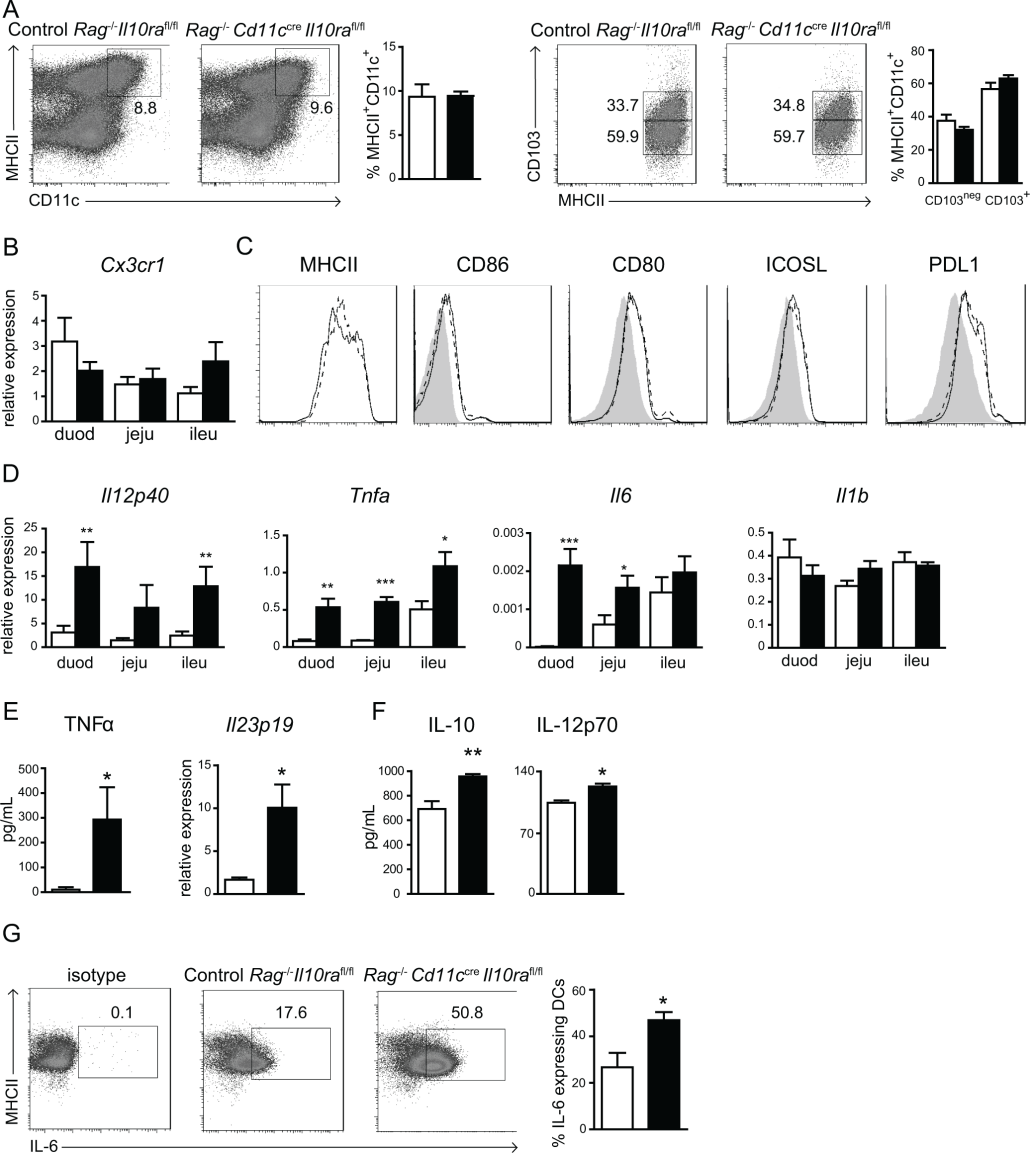
IL-10 control of LP CD11c^+^ cells is necessary to prevent excessive inflammatory cytokine production (**A**) LP cells were stained for MHCII, CD11c and CD103 to evaluate frequencies of CD11c^+^ cell subsets in *Rag^−/−^Cd11c^cre^Il10ra^fl/fl^* and control *Rag^−/−^Il10ra^fl/fl^* mice. Gate on left and right panel: CD45^+^ and CD45^+^MHCII^+^CD11c^+^, respectively. One representative out of 12 mice is shown. (**B**) mRNA expression of *Cx3cr1* was determined in the three parts of the SI as indicated. (**C**) CD11c^+^ cells were stained with antibodies against the indicated surface markers to assess their maturation status. Black lines represent *Rag^−/−^Il10ra^fl/fl^* controls, dotted lines CD11c^+^ cells from *Rag^−/−^Cd11c^cre^Il10ra^fl/fl^* mice and grey peaks the isotype control. One representative animal out of 6 is shown. (**D**) mRNA expression of pro-inflammatory cytokines was determined by quantitative RT-PCR in duodenum (duod), jejunum (jeju) and ileum (ileu) of *Cd11c^cre^Il10ra^fl/fl^* and control *Il10ra^fl/fl^* littermates. mRNA expression is relative to GAPDH. (**E**) LP cells purified from *Rag^−/−^* control *Il10ra^fl/fl^* mice and *Rag^−/−^Cd11c^cre^Il10ra^fl/fl^* mice were stimulated with LPS overnight. TNFα levels were measured in the supernatants and the cells were collected to determine *Il23p19* mRNA expression levels by quantitative RT-PCR relative to GAPDH. (**F**, **G**) LP cells from *Rag^−/−^* control *Il10ra^fl/fl^* mice and *Rag^−/−^Cd11c^cre^Il10ra^fl/fl^* mice were stimulated with LPS and sCD40L overnight. (F) Cytokine levels were measured in the supernatants and (G) intracellular FACS staining for IL-6 was performed. Gate: CD45^+^CD11c^+^. (A) *n* = 12 mice, (B, C) *n* = 6 mice per group, one out of 2 experiments is shown. (D) *n* = 6 mice, one out of 2 experiments is depicted. (E–G) *n* = 3 mice, one out of 3 experiments is shown. Data are represented as mean ± SEM. □ Control *Rag^−/−^Il10ra^fl/fl^*, ■ *Rag^−/−^Cd11c^cre^Il10ra^fl/fl^* littermates.

Next we investigated how the lack of IL-10 signaling in CD11c^+^ cells mediates an enhanced memory T cell response in the SI *in situ*. We previously reported that absent IL-10 signaling does not alter DC homeostasis and maturation in lymphoid tissues and the skin in the steady state [16]. However, IL-10 signals may be critical to maintain resident LP CD11c^+^ cell homeostasis in the SI. In particular, an increased occurrence of CX3CR1^+^CD103^−^ at the expense of CD103^+^ DC in the LP induces intestinal inflammation [22]. Thus, we first assessed the frequencies of total CD11c^+^ cells as well as the CD103^+^ and CD103^−^ subsets and their maturation status in the SI. In *Cd11c^cre^Il10ra^fl/fl^* animals, the frequency of total CD11c^+^ cells was comparable to littermate controls (Figure S4). In order to assess the intrinsic function of the resident intestinal population we crossed the *Cd11c^cre^Il10ra^fl/fl^* mice onto a Rag2-deficient (*Rag^−/−^*) background. As expected, in the absence of T and B cells these *Rag^−/−^Cd11c^cre^Il10ra^fl/fl^* mice exhibited no small intestinal inflammation (data not shown). In addition, the frequency of total CD11c^+^MHCII^+^ cells and subpopulations of CD103^+^ and CD103^−^ cells were similar to *Rag^−/−^Il10ra^fl/fl^* controls (Figure 5A). Moreover, in the duodenum, jejunum and ileum *Cx3cr1* mRNA expression was similar between *Rag^−/−^Cd11c^cre^Il10ra^fl/fl^* and control *Rag^−/−^Il10ra^fl/fl^* mice (Figure 5B) and the CD11c^+^ cells expressed comparable surface levels of MHCII and co-stimulatory molecules (Figure 5C). These data demonstrate that defective IL-10 signaling does not alter the composition of the LP resident CD11c^+^ cell populations nor their phenotypic maturation.

In contrast, throughout the SI mRNA expression of the pro-inflammatory cytokines *Il12p40*, *Tnfa* and *Il6* were elevated in *Cd11c^cre^Il10ra^fl/fl^* mice as determined by quantitative RT-PCR, while mRNA levels of *Il1b* were similar to controls (Figure 5D). These results suggest that IL10Rα-deficient CD11c^+^ cells secrete increased amounts of pro-inflammatory cytokines in the SI. To confirm that this was a CD11c^+^ cell intrinsic effect, we cultured resting LP cells isolated from *Rag^−/−^* animals overnight in the presence of LPS with or without soluble CD40L (sCD40L). Stimulation with LPS alone significantly increased TNFα release by *Rag^−/−^Cd11c^cre^Il10ra^fl/fl^* LP cells as compared to controls (Figure 5E), while other inflammatory proteins such as IL-23, IL12p70 and IL-1β were not detectable (data not shown). However, LPS-stimulated LP cells from *Rag^−/−^Cd11c^cre^Il10ra^fl/fl^* mice expressed significantly more *Il23* mRNA (Figure 5E). These data suggest that IL-10Rα-deficient CD11c^+^ LP cells are more easily activated than control cells. However, T-cell–phagocyte interaction may elicit a more substantial inflammatory response by LP CD11c^+^ cells. In agreement, after incubation with both LPS and sCD40L a small, yet statistically significant, increase in IL12p70 was detected in supernatants of *Rag^−/−^Cd11c^cre^Il10ra^fl/fl^* as compared to *Rag^−/−^Il10ra^fl/fl^* control cultures (Figure 5F). IL-23 and IL-1β protein were not detectable in these LPS and sCD40L stimulated cultures (not shown). Notably, IL-10Rα-deficient CD11c^+^ cells secreted significantly higher levels of IL-10 (Figure 5F). Moreover, intracellular staining of LPS- and sCD40L-stimulated CD11c^+^MHCII^+^ cells revealed that significantly more IL-10Rα–deficient CD11c^+^ cells secreted IL-6 as compared to control cells (Figure 5G). Taken together, these data demonstrate that activated IL-10Rα-deficient CD11c^+^ LP cells produce higher levels of pro-inflammatory cytokines in the SI that could promote the enhanced effector/memory T cell response *in situ*. Moreover, the increased production of IL-10 by activated IL-10Rα-deficient LP CD11c^+^ cells is unable to neutralize the effect of the elevated pro-inflammatory cytokines *in vivo*.

### *Helicobacter hepaticus* elicits inflammation in the large intestine of *Cd11c^cre^Il10ra^fl/fl^* mice

**Figure 6:**
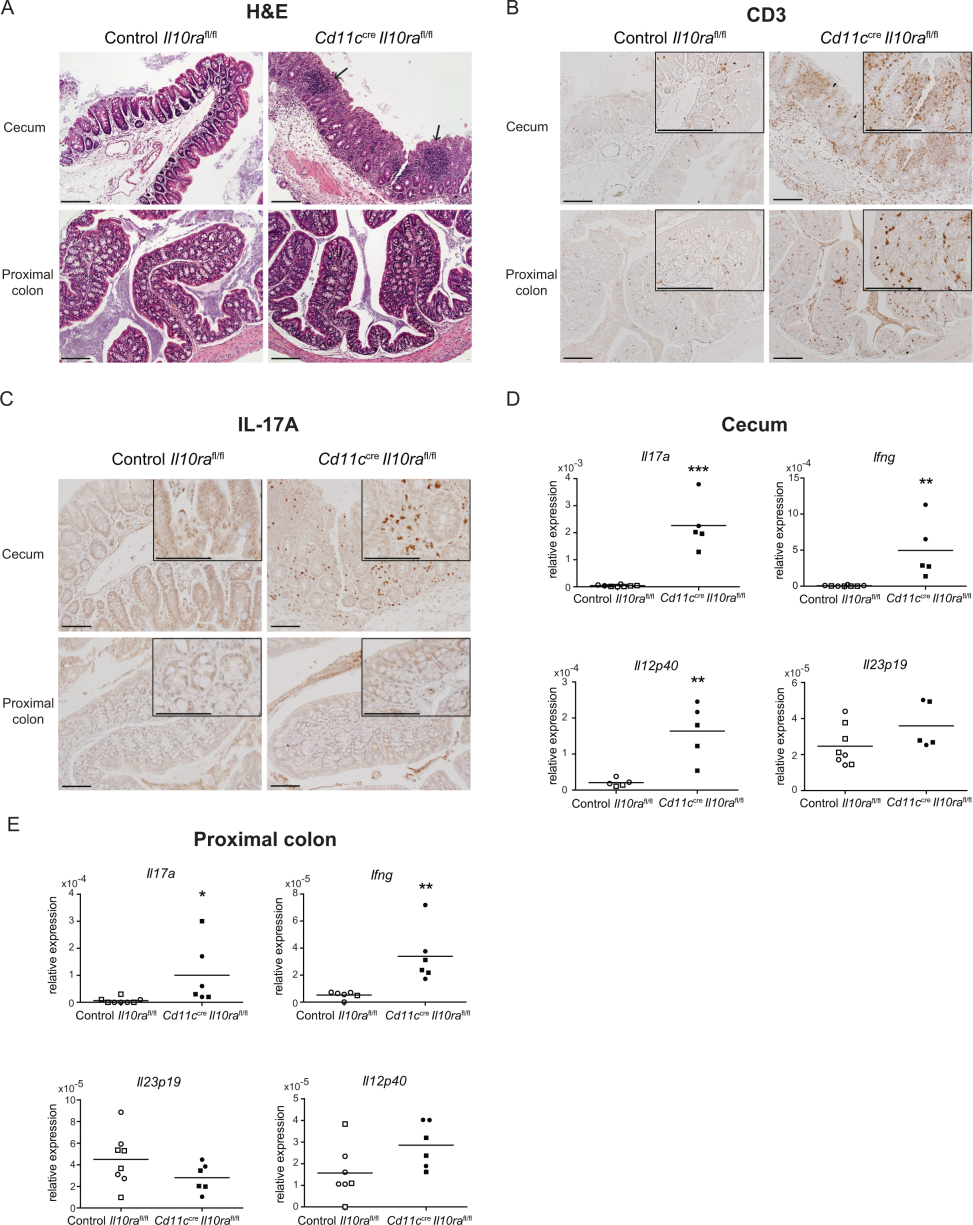
*Cd11c^cre^Il10ra^fl/fl^* mice develop colitis upon colonization with *Helicobacter hepaticus* Twenty-two to 32 week-old control *Il10ra^fl/fl^* and *Cd11c^cre^Il10ra^fl/fl^* littermates were administered *Helicobacter hepaticus* by oral gavage on 3 alternate days. Mice were sacrificed at 6 weeks (○/● for control *Il10ra^fl/fl^* and *Cd11c^cre^Il10ra^fl/fl^*, respectively) or 13 weeks (□/■ for control *Il10ra^fl/fl^* and *Cd11c^cre^Il10ra^fl/fl^*, respectively) after the first administration of *Helicobacter hepaticus*. (**A**, **B**, **C**) 13 weeks after *Helicobacter hepaticus* inoculation, paraffin sections of cecum and proximal colon from control *Il10ra^fl/fl^* and *Cd11c^cre^Il10ra^fl/fl^* mice were stained with (A) H & E (arrows indicate infiltrates), (B) anti-CD3 or (C) anti-IL-17A. (**D**, **E**) mRNA expression levels of cytokines specific of a Th1 or Th17 immune response were measured in sections of cecum and proximal colon from control *Il10ra^fl/fl^* and *Cd11c^cre^Il10ra^fl/fl^* littermates. mRNA is relative to cyclophilin. Data are from *n* = 7–8 mice per group. For histology, one representative animal out of 7–8 is depicted.

As our IVC unit was free of *Helicobacter hepaticus*, which has been directly linked to the induction of large intestinal inflammation in SPF *Il10^−/−^* mice [4, 46], we assessed whether IL-10–mediated control of CD11c^+^ cells was required to suppress *Helicobacter hepaticus*-elicited colitis. To this aim, mice were colonized on three alternate days with ~10^8^ CFU of *Helicobacter hepaticus*. Upon sacrifice 6 or 13 weeks later, *Cd11c^cre^Il10ra^fl/fl^* mice had developed severe large intestinal inflammation, which was most abundant in the cecum and was associated with the infiltration of CD3^+^ cells in the submucosa (Figure 6A and 6B). IL-17A secreting cells were only detected in the cecum and to a lesser extent in the proximal colon of *Cd11c^cre^Il10ra^fl/fl^* animals (Figure 6C). Moreover, mRNA expression of *Ifng* and *Il17a* was significantly increased in cecum and proximal colon segments of colonized *Cd11c^cre^Il10ra^fl/fl^* as compared to control mice (Figure 6D and 6E). To assess whether IL-12, IL-23, IL-6 and/or IL-1β contribute to the inflammatory T cell response we determined mRNA expression of these cytokines in the same tissue segments. In particular, *Il12p40* expression was significantly enhanced both in the cecum and proximal colon (Figure 6D and 6E), while no difference in *Il23p19* (Figure 6D and 6E), *Il6* and *Il1b* mRNA expression was observed (data not shown). In conclusion, these data demonstrate that lack of IL-10Rα signaling in CD11c^+^ cells is sufficient to render mice susceptible to *Helicobacter hepaticus* induced Th1/Th17-mediated large intestinal inflammation.

## DISCUSSION

In this study, we discovered that IL-10–mediated control of CD11c^+^ cells is pivotal for small intestinal but less essential for colonic tolerance under SPF conditions. This is evidenced by the presence of mucosal inflammation and pathology in the SI but not in the colon. Our data highlight the fact that direct IL-10 control of effector/memory T cells and Treg is not sufficient to maintain immune homeostasis in the SI. Instead, indirect control of T cells by LP CD11c^+^ cells that require conditioning via IL-10 *in situ* appears to be crucial. In particular, since naive T cell priming by IL-10Rα–deficient CD11c^+^ cells is not amplified and oral tolerance to harmless protein feed is maintained. Upon increased bacterial pressure, i.e. colonization with *Helicobacter hepaticus*, IL-10 signaling in CD11c^+^ cells is also required to maintain large intestinal homeostasis by suppressing pathogen-driven Th1 and Th17 responses.

Our findings establish that IL-10–mediated regulation of CD11c^+^ cells is required for small intestinal homeostasis. In the absence of IL-10 signaling in CD11c^+^ cells the mice develop classical small intestinal pathology with crypt hyperplasia and increased numbers of iELs. Inflammation was associated with an approximately 20-fold expansion of IL-17A–producing LP cells and enhanced cytokine release by IFNγ^+^ cells, despite enhanced expression of *Il10* mRNA in the LP and secretion of elevated amounts of IL-10 by IL-10Rα–deficient LP CD11c^+^ cells and T cells upon *in vitro* stimulation. The increase in numbers of IL-17A–secreting cells in the presence of elevated levels of IL-10 was unexpected since activated Th17 cells express the IL-10R and need to be controlled by Tr1-derived IL-10 [47]. Strikingly, although the number of Foxp3^+^ cells was unaltered in *Cd11c^cre^Il10ra^fl/fl^* mice, these cells appear unable to inhibit inflammation. This may be explained by the observation that Foxp3^−^ Tr1 regulatory cells constitute the main source of IL-10 in the SI [48, 49]. In agreement, Foxp3^+^ Treg-specific *Il10*^−/−^ mice fail to develop small intestinal inflammation, suggesting that Foxp3^+^ Treg are indeed not the main IL-10–secreting regulatory T cell population in the SI. However, our results prove that there is enough IL-10 available in the SI of *Cd11c^cre^Il10ra^fl/fl^* mice to sustain Treg and curtail memory/effector T cells in the LP. Intriguingly, T cell-specific IL-10–mediated regulatory circuits alone are not sufficient to prevent the development of inflammation. Hence, IL-10–mediated regulation of T cell responses in the SI critically depends on indirect control by CD11c^+^ cells *in situ*. Notably, the phenotype observed in *Cd11c^cre^Il10ra^fl/fl^* mice is indeed CD11c^+^ cell-mediated, since we have previously established that T cells from these mice express physiologic levels of the IL-10Rα and fail to induce inflammation upon adoptive transfer into wild type hosts [16].

Multiple mechanisms may account for the increased numbers and hyper-activated phenotype of LP effector/memory cells. For one, most of the LP T cells in the SI of *Cd11c^cre^Il10ra^fl/fl^* mice expressed Ki67 and fewer cells exhibited surface Annexin V, indicating that CD4^+^ and CD8^+^ T cells are highly proliferative and less apoptotic as compared to control *Il10ra^fl/fl^* mice. Moreover, the increased expression of chemokines, i.e. CCL5, contributes to the attraction of T cells to the site of inflammation. There are multiple functional changes in IL-10Rα–deficient CD11c^+^ cells that may explain these events: IL-10Rα–deficient LP CD11c^+^ cells in the SI expressed elevated levels of the pro-inflammatory cytokines TNFα, IL-6, IL-23 and IL-12p70 upon *in vitro* stimulation with LPS ± sCD40L. In agreement, during *Heliobacter hepaticus* infection enhanced mRNA expression of *Il12p40* and a trend towards increased *Il23p19* were detected in the large intestine. IL-23 is dispensable during naïve T-cell priming, but crucial for the maintenance and reactivation of the memory Th17 pool [50]. In the colon, IL-23 directly acts on T cells to promote inflammation in mice [51]. Moreover, IL-6 plays a major role in maintaining memory T cells [52]. Thus, the enhanced secretion of IL-6 and IL-23 by IL-10Rα–deficient CD11c^+^ cells could directly promote the survival, proliferation and activation of Th17 cells in the SI. TNFα has not been identified as a specific memory T-cell activation marker but as a general pro-inflammatory mediator TNFα may affect both innate and adaptive immune activation. Beyond promoting the differentiation of Th1 cells, which was similar in MLN of *Cd11c^cre^Il10ra^fl/fl^* and control mice, IL-12 has been suggested to play a role in sustaining Th1 cells [53], which may explain their enhanced survival in the SI of *Cd11c^cre^Il10ra^fl/fl^* animals. In conclusion, IL-10Rα–deficient CD11c^+^ cells drive inflammation in the SI through re-activation of Th1 and, in particular, Th17 cells *in situ*, which highlights their role as innate tissue-resident immune regulators.

LP CD11c^+^ cells consist of subpopulations that play distinct roles upon exposure to different types of antigen. Multiple subsets of DC migrate to draining LN to induce differentiation of regulatory or inflammatory effector cells. In particular CD103^+^ DC have been reported to induce conversion of naive T cells into Foxp3^+^ Treg in the steady state [54]. A recent study showed that IRF4 dependent CD103^+^CD11b^+^ DC induce Th17 cell differentiation in the MLN [23]. On the other hand, CX3CR1^+^CD11c^+^ cells have also been reported in the gut draining lymph [26] and IL-10 production by LP CX3CR1^+^ cells is required for the expansion of mucosal Treg and the induction of oral tolerance [12, 31]. To this end, our data do not suggest a differential regulation and most likely all LP phagocyte populations need to be tightly controlled by IL-10. Indeed similar to our study it has recently been shown that specific deletion of the *Il10ra* in CX3CR1^+^ cells elicits spontaneous colitis in a *Helicobacter* positive facility [32]. Hence, the CX3CR1^+^CD11c^+^ population may be responsible for the enhanced susceptibility to *Helicobacter hepaticus*-induced inflammation in the large intestine in our *Cd11c^cre^Il10ra^fl/fl^* mice. However, it was not reported whether mice with IL-10Rα–deficient CX3CR1^+^ cells exhibit small intestinal pathology and whether colonic inflammation in these animals disappears in the absence of *Heliobacter hepaticus*. Therefore, future CD11c^+^ cell subset-specific gene targeting approaches are needed to dissect any differential role of IL-10 in governing distinct DC and macrophage populations in the intestine.

Our data indicate that IL-10 signaling in CD11c^+^ cells is dispensable during priming of naive T cells in the MLN, while IL-10 control of CD11c^+^ cells is crucial during an effector/memory T-cell response in the LP. Moreover, oral tolerance, as determined by inhibition of a systemic delayed type hypersensitivity (DTH) response after harmless protein feed, which is dependent on the migration of DC and subsequent differentiation of functional Treg in MLN [42, 43], is intact in *Cd11c^cre^Il10ra^fl/fl^* mice. Following *in vitro* stimulation IL-10R–deficient LP CD11c^+^ cells secreted elevated levels of pro-inflammatory cytokines, most likely mediating enhanced reactivation of LP CD4^+^ and CD8^+^ T cells and iELs in the absence of exacerbated T-cell priming in MLN of *Cd11c^cre^Il10ra^fl/fl^* mice. Moreover, *in vitro* re-stimulated LP T cells and iELs isolated from *Cd11c^cre^Il10ra^fl/fl^* animals produced increased amounts of IL-2, a cytokine that is essential for T-cell proliferation and survival [44]. These data corroborate our previous report of unaltered and exaggerated T-cell priming and reactivation, respectively, by IL-10R–deficient CD11c^+^ cells during an inflammatory skin reaction [16]. In conclusion, IL-10 appears to be dispensable to govern their classic antigen presenting function in draining LN, but emerges as an essential regulator of the innate function of CD11c^+^ cells to restrain local immune responses and tissue inflammation.

The accumulation of CD4^+^CD8α^−^ and CD4^+^CD8α^+^ iELs was a striking feature of disease in *Cd11c^cre^Il10ra^fl/fl^* mice. All subtypes of iELs isolated from *Cd11c^cre^Il10ra^fl/fl^* mice secreted elevated amounts of IL-17A and IFNγ and less IL-10 upon *in vitro* re-stimulation, which points towards a pro-inflammatory phenotype of these cells. In fact, colonic CD4^+^ iELs of *Il10*^−/−^ mice secrete elevated levels of IL-17A during colitis, which is enhanced by IL-23 [55]. Since IL-10Rα deficient LP DC secrete increased amounts of IL-23, this may account for the enhanced production of IL-17A by iELs in the SI. One intriguing observation is that despite the increased number of IFNγ- and IL-17A–producing iELs, no necrosis of epithelial cells was noticeable in *Cd11c^cre^Il10ra^fl/fl^* animals. In agreement, *Cd11c^cre^Il10ra^fl/fl^* mice did not succumb to a lethal wasting disease due to the local inflammation and even after disruption of the epithelial barrier by EtOH gavage, we failed to detect any bacteria in the serum nor did this treatment elicit systemic inflammation (data not shown).

The finding that lack of IL-10 signaling in LP CD11c^+^ cells does not cause spontaneous large bowel inflammation under SPF conditions was unexpected. Total, T cell-specific and Foxp3^+^ Treg-specific *Il10*^−/−^ mice all develop colonic inflammation [11]. Moreover, IL-10 control of Foxp3^+^ Treg is necessary to maintain immune homeostasis in a colitis transfer model [9, 12]. These data suggest that IL-10 signaling in Foxp3^+^ Treg might be sufficient, whereas IL-10 control of CD11c^+^ cells may play a less important role to maintain immune homeostasis in the colon under SPF conditions. On the other hand, mice with defective IL-10 signaling in intestinal macrophages develop spontaneous colitis when bred in a *Helicobacter*-positive facility [32], but are free of spontaneous colonic inflammation if *Helicobacter* spp. are absent [34, 35]. In agreement, once the *Cd11c^cre^Il10ra^fl/fl^* mice were colonized with *Helicobacter hepaticus*, large intestinal inflammation associated with increased Th1 and Th17 responses developed. The differential regulation of LP CD11c^+^ cells by IL-10 might be linked to either their anatomical location and/or the differences in exogenous antigens that predominate at these sites [49]. In the SI, a layer of epithelial cells separates the LP from the environment, such that CD11c^+^ cells are constantly in close contact with luminal antigens. On the other hand, the epithelial layer of the colon is protected by a thick mucus coating, which provides a much stronger physical barrier and hinders antigens from penetrating the epithelium. While food antigens dominate in the proximal part of the SI, more distally commensal microbiota account for the majority of the antigenic load. In fact, celiac disease patients, who develop a specific reactivity to the food antigen gluten, exhibit duodenal pathology but no colonic inflammation [36]. Crohn's disease, occurring in terminal ileum and/or colon, and ulcerative colitis, a colonic disease, are both associated with inflammatory immune reactions to commensal microbiota that predominate at these intestinal locations. Intriguingly, in *Cd11c^cre^Il10ra^fl/fl^* mice the excessive expression of pro-inflammatory Th1 and Th17 cytokines decreased from duodenum to ileum, which further highlights the fact that IL-10 control of CD11c^+^ cells might be crucial to curtail memory responses to food rather than microbial antigens. On the other hand, although the particular component of the microbiota remains unknown, the specific increase in IL-17–producing T cells and exacerbated response to *Helicobacter hepaticus* colonization argue in favor of a bacteria-driven memory T-cell response in the inflamed SI of *Cd11c^cre^Il10ra^fl/fl^* mice. Hence, distinct IL-10–mediated regulatory pathways likely govern different CD11c^+^ cell responses to particular types of luminal antigens.

In conclusion, the phenotype of *Cd11c^cre^Il10ra^fl/fl^* mice exhibits features of intestinal inflammation that are also seen in human disease. In particular, in celiac disease duodenal inflammation is characterized by crypt hyperplasia, increased numbers of iELs and IgA^+^ plasmablasts as well as enhanced levels of Th1/Th17 cytokines. The elevated production of pro-inflammatory cytokines by IL-10Rα–deficient CD11c^+^ cells provokes the enhanced recruitment and activation of LP T cells and iELs. Moreover, we establish a critical role of LP CD11c^+^ cells and their control by the anti-inflammatory cytokine IL-10 to maintain immune homeostasis in the SI.

## MATERIALS AND METHODS

### Mice

CD11c-Cre were crossed to IL-10Rα^fl/fl^ mice to obtain *Cd11c^cre^Il10ra^fl/fl^* animals (see Supplementary References). *Cd11c^cre^Il10ra^fl/fl^* were crossed to Rag2-deficient mice (Jackson Laboratories) to generate *Rag^−/−^Cd11c^cre^Il10ra^fl/fl^* double knockout animals. All mice were on a C57BL/6 background. All experiments were performed on age-matched littermates to ensure similar microbiota between animals. Mice were sacrificed at the age of 20 weeks or older, unless stated otherwise in the figure legend. Mice were kept under specific pathogen free (SPF) conditions in the animal facility of the Erasmus MC. All animal experimentation was in compliance with European Union and national laws and approved by the local ethical committee.

### LP T cell and iEL isolation

Whole SI and colon tissue was cut and incubated twice in HBSS (Gibco) supplemented with 10% FCS (Integro), 5 mM EDTA (Sigma) and 1 mM DTT (Sigma) for 20 min at 37°C. The supernatant containing iELs was washed and resuspended in 44% percoll (Pharmacia), overlayered with 67% percoll and centrifuged for 20 min at 1800 rpm. iELs were harvested from the interface layer. The precipitate, containing the LP cells, was digested in RPMI supplemented with 10% FCS, 100 U/mL collagenase type VIII (Sigma) and 0.01 mg/mL DNAse (Sigma). Cells were passed through a 70 μm nylon cell strainer and resuspended in 100% percoll, which was overlayered with 40% percoll, and centrifuged for 20 min at 1800 rpm. LP cells were recovered from the interface layer.

### Cell culture and adoptive T cell transfer

OTII mice were sacrificed, spleen and peripheral LN isolated and mashed through a 70 μm filter. OTII cells were sorted using MACS microbeads (CD4^+^ T cell isolation kit, Miltenyi Biotec) and stained with 0.5 μM CFSE (Molecular Probes) to follow their division.

For *in vitro* CD11c^+^ cell-T cell coculture, CD11c^+^ cells were isolated from MLN, stained with CD11c-PE and sorted using MACS microbeads (anti-PE microbeads, Miltenyi Biotec). 1 × 10^4^ CD11c^+^ cells were plated with 1 × 10^5^ OTII T cells in complete IMDM (10% FCS, 2 mmol/L L-glutamine, 100 μg/mL streptomycin, 100 U/mL penicillin and 50 μmol/L β-mercaptoethanol) in the presence of 0.5 ug/mL of OVA protein (Sigma). After three days, supernatants were collected for cytokine measurements and cells were analyzed by flow cytometry for CFSE dilution.

For *in vivo* experiments, on day 0, 10 × 10^6^ OTII cells were injected i.v. into naïve recipient mice. On day 1, mice were gavaged with 70 mg OVA protein, and on day 4, animals were sacrificed and MLN collected for analysis.

### DC and T cell restimulation *in vitro*

To measure T cell derived cytokines, LP cells were incubated at 1 × 10^6^ cells per mL in complete IMDM supplemented with 0.05 μg/mL PMA and 0.5 μg/mL ionomycin (both from Sigma) in the presence of 1 μg/mL Golgistop (BD). Notably, restimulation cultures were not corrected for the number of T cells but for the number of *total* LP cells.

To determine DC-derived cytokines, LP cells from Rag^−/−^ animals were incubated with 0.5g/mL LPS (Sigma) with or without 400 ng/mL sCD40L (Peprotech) overnight at 37°C. Supernatants were collected to measure cytokines and cells were harvested for mRNA isolation.

### Additional methods

Antibodies, flow cytometry, cytokine detection, quantitative PCR, histology and *Helicobacter hepaticus* colonization are described in the Supplementary Methods.

### Statistics

Student *t*-test was used to compare control *Il10ra^fl/fl^* to *Cd11c^cre^Il10ra^fl/fl^* mice. *p < 0.05, **p < 0.01 and ***p < 0.001 throughout the manuscript.

## SUPPLEMENTARY MATERIAL FIGURES AND TABLES


