# Supplementary material for: IL-10 control of CD11c+ myeloid cells is essential to maintain immune homeostasis in the small and large intestine
Source: Oncotarget. 2016 Mar 24;7(22):32015–30. doi: 10.18632/oncotarget.8337 (PMC5077993; doi:10.18632/oncotarget.8337)
Supplement: Supplementary file 1 [file oncotarget-07-32015-s001.pdf]

IL-10 control of CD11c$^+$ myeloid cells is essential to maintain immune homeostasis in the small and large intestine

Supplementary Materials

SUPPLEMENTARY MATERIAL AND METHODS

Flow cytometry

mLN, LP cells and iELs were stained with the following anti-mouse antibodies from Biolegend: CD45 (30-F11), CD3 (145-2C11), CD4 (GK1.5), CD8α (53-6.7), B220 (RA3-6B2), MHCII (M5/114.15.2), CD11c (N418), CD103 (2E7), CD86 (GL-1), CD80 (16-10A1), PD-L1 (MIH5), ICOSL (HK5.1) and CD25 (3C7). The eBioscience Foxp3 (FJK-16s) kit was used to stain Foxp3$^+$ Treg according to the manufacturer's protocol. For intracellular staining, cells were fixed in PFA 2% and subsequently stained in 0.25% saponine buffer with the following anti-mouse antibodies from Biolegend: IL-17A (TC11-18H10.1), IFNγ (XMG1.2), IL-2 (JES6-5H4) and IL-6 (MP5-20F3). Appropriate isotype controls were used when necessary. Cells were analyzed on a FACS Canto (BD) with FlowJo software.

Quantitative RT-PCR

mRNA was extracted from cells using the GenElute mammalian total RNA miniprep kit (Sigma-Aldrich). cDNA was synthesized from mRNA with SuperScript II reverse transcriptase (Invitrogen), according to the manufacturer's protocol. All reactions were run on a 7900 HT Fast Real Time PCR machine or on a AbiPrismR7900 Sequence Detection System (Applied Biosystems). Primer sequences are listed in Supplementary Table 1.

Cytokine detection

Concentrations of IL-2, IL-4, IL-6, IL-10, IL-12p70, IL-17A, IL-22, IFNγ, TNFα in supernatants were determined by Cytometric Bead Array (CBA) according to the manufacturer's instructions (BD).

Histology

Small intestinal and colonic tissue was fixed in 4% PFA and paraffin embedded. 4 µm sections were stained with hematoxylin (Vector Laboratories) and eosin (Sigma) or Alcian blue 0.1%, Periodic acid 1% and Schiff's (PAS) reagent (all from Sigma). For immunohistochemical detection of CD3 (Dako Heverlee), Ki67 (Novocastra), IL-17A (Biolegend), BrdU (Roche) and IgA (Acris Antibodies), endogenous peroxidases were quenched with 3% H_2O_2 in methanol for 20 min. Microwave treatment in citrate buffer (10 mM, pH 6.0) was performed for antigen retrieval. Blocking was done for 1 h in 10 mM Tris, 5 mM EDTA, 0.15 M NaCl, 0.05% Tween-20 and 10% normal mouse serum. Antibody incubation was overnight at 4°C. Immunoreactions were detected with biotinylated secondary goat anti-rabbit serum using the Vectastain ABC Elite Kit (Vector Laboratories) and 3,3′-diaminobenzidine tetrahydrochloride (Sigma-Aldrich). Sections were counterstained with hematoxylin.

In several experiments ethanol (EtOH) gavage was used to breach the intestinal epithelial barrier [1]. Thereto, the mice were gavaged with 150 µl of 50% EtOH on two consecutive days, sacrificed 72 h after the first gavage and analyzed by immunohistochemistry.

Helicobacter hepaticus colonization

Helicobacter hepaticus was grown under microaerophilic conditions as previously described [2]. *Helicobacter hepaticus* viability was confirmed using a bacterial live/dead kit (BacLight, Invitrogen). Mice were colonized with *Helicobacter hepaticus* (~10^8 CFU) by oral gavage three times on alternate days and sacrificed 6 or 13 wk after the last inoculation.

Oral tolerance induction

For oral tolerance induction mice received 25 mg ovalbumin (OVA) in 200 µL saline by oral gavage to induce tolerance (+OVA feed). Control groups (−OVA feed) were left untreated. On day 3, the mice were sensitized with 100 µg of $OVA_{323–339}$ peptide in CFA by sub-cutaneous (s.c.) injection in the tail base. On day 8, mice were anesthetized and challenged by intra-dermal (i.d.) injection of 15 µg of $OVA_{323–339}$ into the ear. On day 9, mice were anesthetized and ear thickness was measured using a micrometer. Delayed type hypersensitivity (DTH) responses were expressed as the mean increase in ear thickness of both ears following subtraction of ear thickness before challenge. Differences in ear thickness between tolerant and non-tolerant groups were comparable

on day 1–4 after i.d. challenge. In all experiments ear thickness was measured in a blinded fashion.

Additional references regarding mouse breeding

CD11c-Cre mice [3] were crossed to IL10Rα$^{fl/fl}$ mice [4] to obtain *Cd11c*^{cre}*Il10ra*$^{fl/fl}$ mice.

REFERENCES

1. Boirivant M, Amendola A, Butera A, Sanchez M, Xu L, Marinaro M, Kitani A, Di Giacinto C, Strober W, Fuss IJ. A transient breach in the epithelial barrier leads to regulatory T-cell generation and resistance to experimental colitis. Gastroenterology 2008; 135:1612–1623.
2. Hue S, Ahern P, Buonocore S, Kullberg MC, Cua DJ, McKenzie BS, Powrie F, Maloy KJ. Interleukin-23 drives innate and T cell-mediated intestinal inflammation. J Exp Med. 2006; 203:2473–2483.
3. Caton ML, Smith-Raska MR, Reizis B. Notch-RBP-J signaling controls the homeostasis of CD8- dendritic cells in the spleen. J Exp Med. 2007; 204:1653–1664.
4. Pils MC, Pisano F, Fasnacht N, Heinrich JM, Groebe L, Schippers A, Rozell B, Jack RS, Muller W. Monocytes/macrophages and/or neutrophils are the target of IL-10 in the LPS endotoxemia model. Eur J Immunol. 2010; 40:443–448.

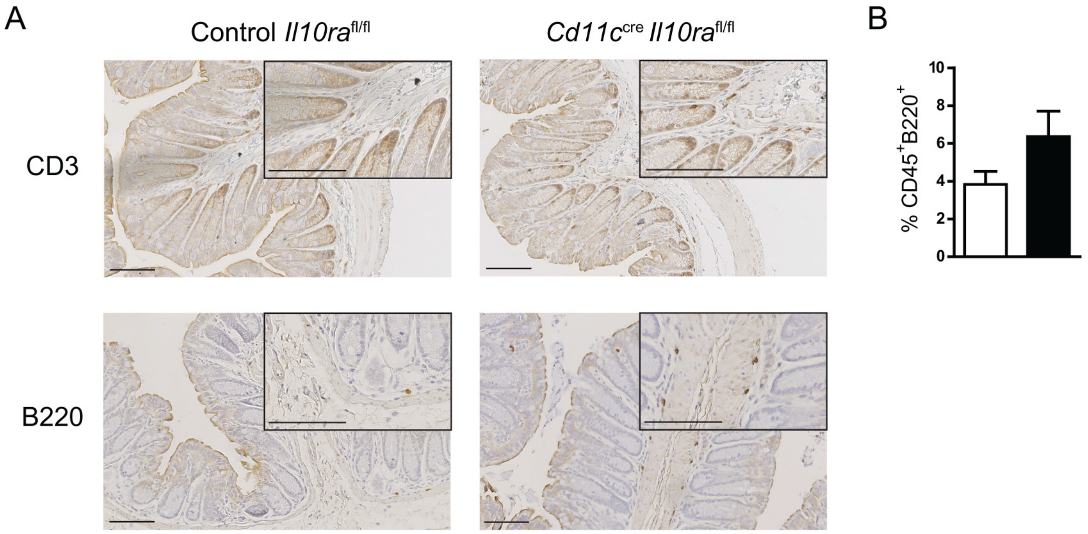

Supplementary Figure S1: Colonic immune homeostasis is preserved in *Cd11c*^{cre}*Il10ra*$^{fl/fl}$ mice under SPF conditions. (**A**) Sections of colon were stained with antibodies against CD3 and B220. One representative mouse out of 12 is depicted. Bar represents 100 µm. (**B**) LP cells were isolated from colon and stained with CD45 and B220 to assess the frequency of B cells. One out of 3 experiments with *n* = 4 mice per group is depicted. Data are represented as mean ± SEM. □ Control *Il10ra*$^{fl/fl}$ mice, ■ *Cd11c*^{cre}*Il10ra*$^{fl/fl}$ mice.

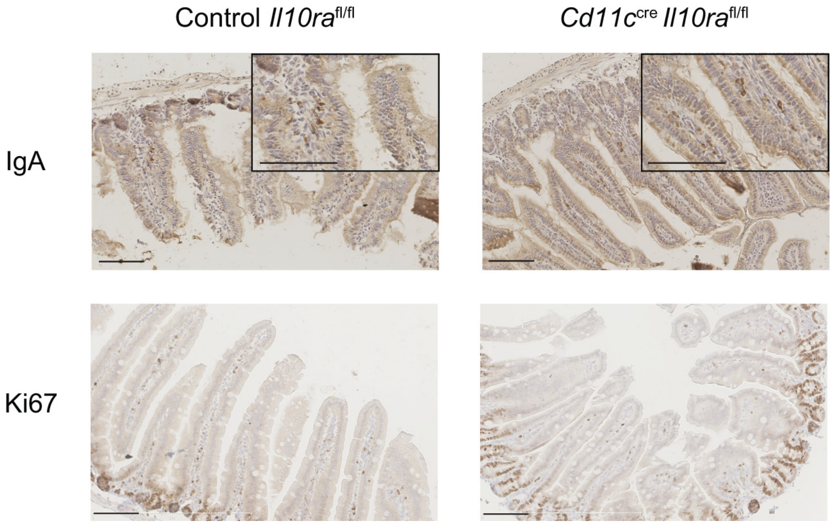

Supplementary Figure S2: Immune homeostasis in young *Cd11c*^{cre}*Il10ra*$^{fl/fl}$ mice is not perturbed. Duodenum sections of 6 week-old *Cd11c*^{cre}*Il10ra*$^{fl/fl}$ and control *Il10ra*$^{fl/fl}$ animals were stained with anti-IgA and anti-Ki67 antibodies as indicated. Bar represents 100 µm. One representative animal out of 12 is depicted.

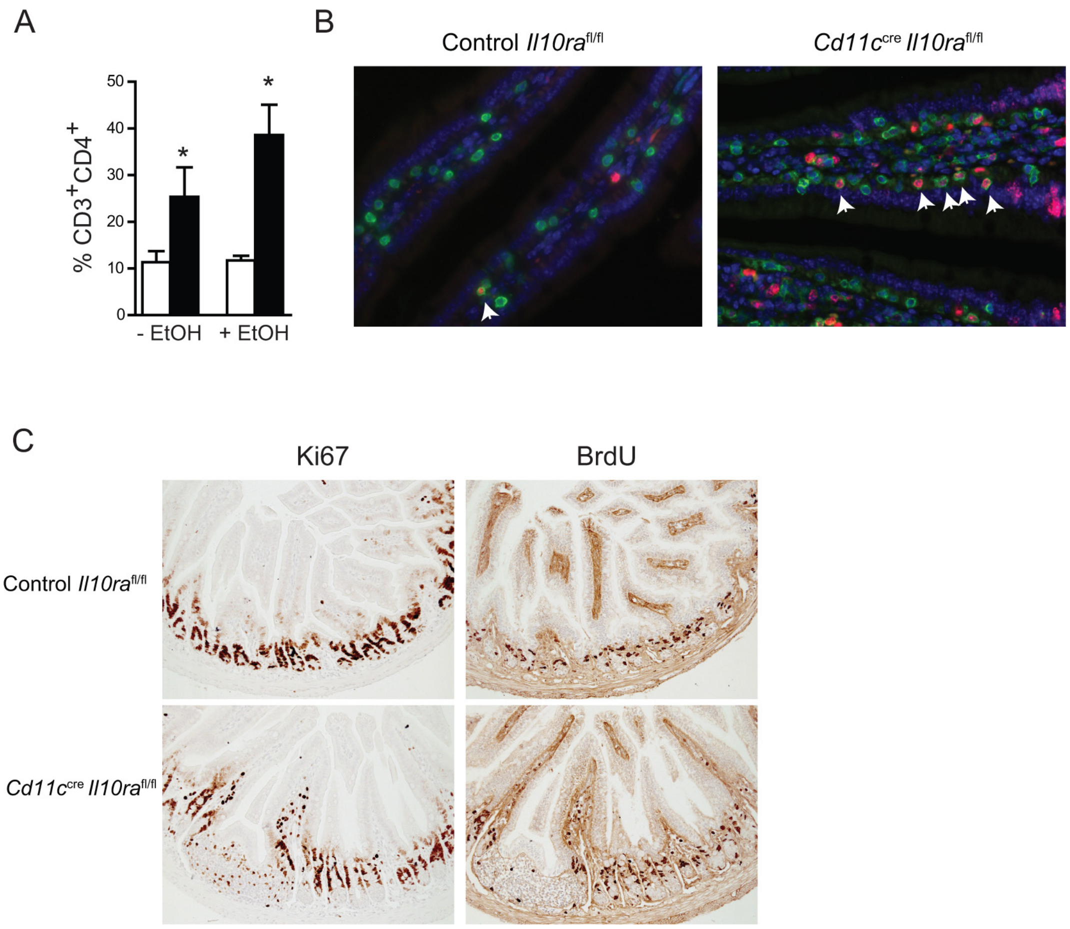

Supplementary Figure S3: Elevated numbers of LP T cells in *Cd11c*^{cre}*Il10ra*$^{fl/fl}$ mice. (**A**) Animals were gavaged on day 0 and day 1 with 50% EtOH and sacrificed on day 2. LP cells were isolated and stained with CD45, CD3 and CD4 to evaluate the frequency of CD4^{+} T cells. Gate: CD45^{+} cells. Data are represented as mean ± SEM. □ Control *Il10ra*$^{fl/fl}$, ■ *Cd11c*^{cre}*Il10ra*$^{fl/fl}$ mice. (**B**) Staining with fluorescently labeled anti-CD3 (green) and anti-Ki67 (red) was performed on duodenum sections from untreated 30–33 week-old *Cd11c*^{cre}*Il10ra*$^{fl/fl}$ and control *Il10ra*$^{fl/fl}$ mice. Nuclei were counterstained with DAPI. One representative out of 5 mice is depicted. Arrows indicate CD3^{+}Ki67^{+} cells. (**C**) Untreated 30–33 week-old control *Il10ra*$^{fl/fl}$ or *Cd11c*^{cre}*Il10ra*$^{fl/fl}$ mice received 50 µg/g bodyweight BrdU i.p.. After 2 h mice were sacrificed and duodenum biopsies were paraffin embedded. Serial sections were stained with anti-BrdU or anti-Ki67. One representative out of 5 mice is depicted.

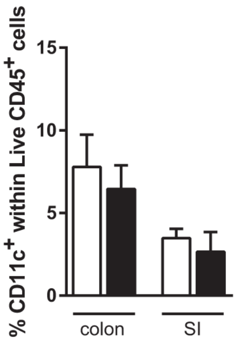

Supplementary Figure S4: Unperturbed frequency of CD11c^{+} cells in *Cd11c*^{cre}*Il10ra*$^{fl/fl}$ mice. Small intestinal and colonic LP cells were isolated from *Cd11c*^{cre}*Il10ra*$^{fl/fl}$ mice and control littermates and stained with CD45 and CD11c to evaluate the frequency of CD11c^{+} cells. Gate: Live CD45^{+} cells. Data are represented as mean ± SEM. □ Control *Il10ra*$^{fl/fl}$, ■ *Cd11c*^{cre}*Il10ra*$^{fl/fl}$ mice. One out of 3 experiments with $n = 3$ mice per group is depicted.

Supplementary Table S1: Quantitative RT-PCR primers

Gene	Forward primer sequence	Reverse primer sequence
Cx3cr1	AAGTTCCCTTCCCATCTGCT	CAAAATTCTCTAGATCCAGTTCAGG
Cyclophilin	AACCCCACCGTGTTCT	CATTATGGCGTGTAAAGTCA
Gapdh	AGCTTGTCATCAACGGGAAG	TTTGATGTTAGTGGGGTCTCG
Ifng	ATCTGGAGGAACTGGCAAAA	TTCAAGACTTCAAAGAGTCTGAGGTA
Il1b	TGTAATGAAAGACGGCACACC	TCTTCTTTGGGTATTGCTTGG
Il6	TCTAATTCATATCTTCAACCAAGAGG	TGGTCCTTAGCCACTCCTTC
Il10	CAGAGCCACATGCTCCTAGA	GTCCAGCTGGTCCTTTGTTT
Il12p40	TGGACTGGACTCCCGATG	CATCTTCTTCAGGCGTGTCA
Il17a	TTTTCAGCAAGGAATGTGGA	TTCATTGTGGAGGGCAGAC
Il21	GACATTCATCATTGACCTCGTG	TCACAGGAAGGGCATTTAGC
Il22	TTTCCTGACCAAACTCAGCA	TCTGGATGTTCTGGTCGTCA
Il23p19	TCCCTACTAGGACTCAGCCAAC	TGGGCATCTGTTGGGTCT
Tnfa	CCACGTCGTAGCAAACCAC	TTTGAGATCCATGCCGTTG
